# A natural language processing approach to modelling treatment alliance in psychotherapy transcripts

**DOI:** 10.1192/bjo.2021.177

**Published:** 2021-06-18

**Authors:** Jihan Ryu, Stephen Heisig, Caroline McLaughlin, Rebeccah Bortz, Michael Katz, Xiaosi Gu

**Affiliations:** Icahn School of Medicine at Mount Sinai

## Abstract

**Aims:**

Patient-therapist alliance is a critical factor in psychotherapy treatment outcomes. This pilot will identify language concepts in psychotherapy transcripts correlating with the valence of treatment alliance using natural language processing tools. Specifically, high-order linguistic features will be extracted through exploratory analysis of texts and interpreted for their power to discriminate alliance rated by patients.

**Method:**

Adult patients and therapists in outpatient clinic at various stages of relationship building and treatment goals consented to participate in the cross-sectional study approved by the Institutional Board Review. Psychotherapy sessions were recorded using wireless microphones and transcribed by two research assistants. After the recording, each patient completed Working Alliance Inventory– Short Form, to generate clinical scores of alliance. We used the Linguistic Inquiry Word Count (LIWC) tool to map words to psycholinguistic categories, and generated novel linguistic parameters describing the individual language for each speaker role. Canonical-correlational analysis and descriptive statistics were used to analyze the two datasets.

**Result:**

Patients (N = 12, 83% female, mean age = 40) were primarily diagnosed with personality disorders (67%) working on real-life interpersonal issues (median treatment duration 18.5 weeks, 50% psychodynamic, 32% cognitive-behavioral, 16% supportive modality). In this heterogenous sample, patients who used the “achieve” (e.g. trying, better, success, failure) and “swear” psycholinguistic categories of words rated the treatment alliance lower (r=−0.70, p = 0.01; r=−0.65, p = 0.02). Patients rated alliance lower with therapists, who used more “I” pronoun (r=−0.58, p < 0.05) and higher with therapists using more “risk” (difficult, safe, crisis) and “power” (important, strong, inferior, passive) categories (r = 0.66, p = 0.02, r = 0.58, p < 0.05), which commonly appeared in psychoeducation and conceptual framing of problems. Interestingly, there was no correlation with “affiliation” category (p = 0.9). Linear regression modeling from “achieve,” “swear” variables and “I,” “risk” variables with duration of treatment as covariate predicted the patient's rating of alliance (Adjusted R2 = 0.66, p = 0.03).

**Conclusion:**

Our data collection and sub-sample analysis are ongoing. Preliminary results are showing speaker-specific language patterns in cognitive-emotional domain, e.g. self-expressivity, and in clinician's therapy style, covarying with the patient's perceived closeness in the heterogenous treatment dyads. Novel application of natural language processing to characterize alliance using the data-driven approach is an unbiased method that can provide feedback to clinicians and patients. This characterization can also potentially provide insights into the mechanisms underlying the therapeutic process and help develop psycholinguistic markers for this critical clinical phenomena.

